# Density‐dependent within‐patch movement behavior of two competing species

**DOI:** 10.1002/ece3.10753

**Published:** 2023-11-20

**Authors:** James T. Cronin, Jerome Goddard, Aaron Krivchenia, Ratnasingham Shivaji

**Affiliations:** ^1^ Department of Biological Sciences Louisiana State University Baton Rouge Louisiana USA; ^2^ Department of Mathematics and Computer Science Auburn University Montgomery Montgomery Alabama USA; ^3^ Department of Mathematics and Statistics University of North Carolina Greensboro Greensboro North Carolina USA

**Keywords:** density‐dependent movement, diffusion, microcosm experiment, spatial spread, *Tribolium castaneum*, *Tribolium confusum*

## Abstract

Movement behavior is central to understanding species distributions, population dynamics and coexistence with other species. Although the relationship between conspecific density and emigration has been well studied, little attention has been paid to how interspecific competitor density affects another species' movement behavior. We conducted releases of two species of competing *Tribolium* flour beetles at different densities, alone and together in homogeneous microcosms, and tested whether their recaptures‐with‐distance were well described by a random‐diffusion model. We also determined whether mean displacement distances varied with the release density of conspecific and heterospecific beetles. A diffusion model provided a good fit to the redistribution of *T. castaneum* and *T. confusum* at all release densities, explaining an average of >60% of the variation in recaptures. For both species, mean displacement (directly proportional to the diffusion rate) exhibited a humped‐shaped relationship with conspecific density. Finally, we found that both species of beetle impacted the within‐patch movement rates of the other species, but the effect depended on density. For *T. castaneum* in the highest density treatment, the addition of equal numbers of *T. castaneum* or *T. confusum* had the same effect, with mean displacements reduced by approximately one half. The same result occurred for *T. confusum* released at an intermediate density. In both cases, it was total beetle abundance, not species identity that mattered to mean displacement. We suggest that displacement or diffusion rates that exhibit a nonlinear relationship with density or depend on the presence or abundance of interacting species should be considered when attempting to predict the spatial spread of populations or scaling up to heterogeneous landscapes.

## Graphical abstract

At present, relatively little is known about how conspecific and interspecific competitor density affect movement behavior. Using two competing species of flour beetle, we found that there is a humped‐shaped relationship between the mean displacement rate of each beetle species and its density. The total density of beetles, both species combined, is more important than species identity in affecting species movement.

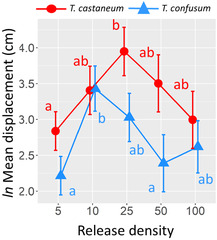

## 1 INTRODUCTION

Species movement behavior can have major consequences for individual fitness, species' distributions, spatial spread, interactions with other species, population dynamics and stability (Chesson, 2000; Hanski, 1999; Hastings, 1978; Holt, 1997; Hubbell, 2001). Considering the important need to predict how populations will respond to present‐day threats from invasive species, habitat loss and fragmentation, and climate change, understanding causes and consequences of movement at both the patch and landscape levels is vital for population management and conservation. Theoretical studies play an extremely important role in predicting population‐level effects of movement (Bowler & Benton, 2005; Turchin, 1998). Early theoretical models on spatial population spread or metapopulation dynamics lacked a great deal of realism, typically treating the rate of movement or dispersal as a constant (e.g., Hanski & Gilpin, 1991; Levins, 1969; Pacala & Roughgarden, 1982; Skellam, 1951). Subsequent models often incorporated a positive linear response to density. Models have largely ignored alternative and nonlinear responses to conspecific density and the conditional dependency of dispersal; for example, presence of interspecific competitors or predators (but see, e.g., Baines et al., 2020; Cronin et al., 2019; Dallas et al., 2020; Harman et al., 2020; Rodrigues & Johnstone, 2014).

For many species across a wide range of taxonomic groups, movement within a relatively homogeneous environment (e.g., within‐patch movement) is well‐described as a simple diffusion process (e.g., Antonelli et al., 1993; Fortin et al., 2013; Kareiva, 1983; Skalski & Gilliam, 2003; Turchin & Thoeny, 1993). Exceptions exist, often attributed to longer or fatter (leptokurtic) tails in the number of individuals recaptured or resighted with distance (e.g., Cronin et al., 2000; Fraser et al., 2001; Hawkes, 2009), but these exceptions can be accommodated in a diffusion framework (e.g., Cronin et al., 2000; Hawkes, 2009; Inoue, 1978; Okubo, 1980; Skalski & Gilliam, 2003; Turchin, 1998). Relatively few studies have explicitly tested whether the rate of within‐patch movement (e.g., diffusion rate, net displacement rate) is dependent on conspecific density and fewer have examined the shape of that relationship; for example, a positive, negative or nonlinear density dependence (Cronin et al., 2001, 2017; Fronhofer et al., 2017; Liu et al., 2011, 2016; Simmons & Thomas, 2004). However, a larger body of literature has addressed the closely related relationship between conspecific density and emigration (for recent review, see Harman et al., 2020). The most obvious expectation is that to alleviate the negative effects of intraspecific competition, movement rates should be positively related to conspecific density (Bowler & Benton, 2005; Matthysen, 2012; Turchin, 1998). Nevertheless, other relationships exist in nature (Harman et al., 2020). Negative density‐dependent movement could arise if there is a benefit to living in a larger group that outweighs the cost of increasing intraspecific competition (Bowler & Benton, 2005; Kim et al., 2009). If resource limitation becomes a problem at higher densities for a gregarious species, movement rates with respect to density could become u‐shaped (Harman et al., 2020). Even humped‐shaped relationships are possible. For example, in small groups, less mobile animals may be less noticeable to predators, while larger groups may be more sedentary because they are more defensible against predators or better capable of acquiring resources (Harman et al., 2020). Different forms of density‐dependent within‐patch movement rates can greatly influence the rate of population spread, encounters with patch boundaries, emigration rates and ultimately metapopulation dynamics.

Very little is known regarding the influence of an interspecific competitor's density on another species' dispersal or movement patterns (Dallas et al., 2019, 2020). This is surprising given that a key mechanism thought to promote coexistence among competing species is a tradeoff between dispersal and competitive ability (Chesson, 2000). Here, poorly competitive but highly dispersive species can coexist with highly competitive but poorly dispersive species at the regional scale because of the spatial variation that arises in their distributions (e.g., Amarasekare, 2003; Chesson, 2000; Kneitel & Chase, 2004; Levins & Culver, 1971; Tilman & Kareiva, 1997). Effectively, the better disperser persists as a fugitive in those habitats that have not yet been colonized by the superior competitor. However, the focus of these studies has been primarily on comparing emigration or colonization rates between species without consideration of the possibility that these rates are dependent on the presence or abundance of the other species (Dallas et al., 2019, 2020). There has been even less focus on interspecific competitor effects on the movement of a species within a patch (Cantrell & Cosner, 1998; Cosner & Lazer, 1984; He & Ni, 2016). However, those movements are likely to influence encounters with the patch boundary and the probability of emigration. Moreover, it has been hypothesized that intraspecific and interspecific competition may influence dispersal of a species differently (Dallas et al., 2020; De Meester et al., 2015; Fronhofer et al., 2017), although this also has rarely been explored (Dallas et al., 2020). In that study by Dallas et al. (2020), *T. castaneum d*ispersal was reduced in the presence of *T. confusum*, while *T. confusum* dispersal was unaffected by the other species. We suggest that theoretical and empirical research, focusing on realistic aspects of animal movement behavior, are needed if we hope to understand how competing species interact and affect their spatial population dynamics and coexistence.

In this study, we conducted a series of microcosm experiments to quantify the effects on conspecific and heterospecific density on the pattern (i.e., fit of a diffusion model to recaptures‐with‐distance) and rate (diffusion rate, net displacement) of movement within a homogeneous environment. We use a model system for competition studies – two species of flour beetles, *Tribolium confusum* and *T. castaneum* (Dallas et al., 2021; Goodnight & Craig, 1996; Hastings & Costantino, 1987; Park, 1948, 1954). We tested the following predictions: (1) the redistribution of male and female flour‐beetles of both species is well described by a diffusion model; (2) the rate of diffusion (or net displacement) is a positive linear function of conspecific density (see Harman et al., 2020); and (3) potential interspecific competitors also have a positive density‐dependent effect on rates of movement.

## 2 METHODS AND MATERIALS

### 2.1 Study system

*Tribolium* flour beetles (Coleoptera: Tenebrionidae) are important worldwide pests of stored flour and grains (Good, 1936). Because of their short generation time (ca. 35 days at 30 C and 50% RH), high fecundity, and simple resource needs, *Tribolium* species have become model systems for the study of species interactions and population dynamics (e.g., Benoit et al., 1998; Dallas et al., 2019; Edmunds et al., 2003; Jillson, 1980; Leslie et al., 1968; Park, 1948, 1954; Yan et al., 1994). In a classic experimental study by Thomas Park (1954), it was demonstrated that competitive superiority between *T. confusum* (confused flour beetle) and *T. castaneum* (red flour beetle) depended on environmental conditions (temperature and humidity). In the years that followed, many other studies of competition between these species, and evolutionary outcomes of this interaction, have been explored (Dallas et al., 2019; Edmunds et al., 2003; Goodnight & Craig, 1996; Korona, 1989; Legault et al., 2020).

*Tribolium* species have also been used to study aspects of animal dispersal and range expansion. The mode of dispersal within a patch (e.g., within a stored‐products facility) is generally via walking, and most dispersal studies with *Tribolium* have focused on this mode of transportation (Campbell et al., 2022; Ridley et al., 2011). However, at least for *T. castaneum*, dispersal among stored‐product facilities often occurs via flight (Daglish et al., 2017; Gurdasani et al., 2018; Ridley et al., 2011). Using a two‐patch system (vials connected by a tube), Ritte and Lavie (1977) found that dispersal ability tended to be greater for adult male than female *T. castaneum* (see also Hagstrum et al., 1998; Ogden, 1970b). However, when selecting for walking distance in *T. castaneum*, Matsumura and Miyatake (2015) found that females were always more mobile than males regardless of whether selection was for longer or shorter walking distances. Ziegler (1977) found that *T. castaneum* has a higher propensity to disperse than *T. confusum* and that the former species exhibits a strong positive density‐dependent emigration response (see also Korona, 1991). In a departure from the highly simplified experimental systems used above, Romero et al. (2009) explored movement behavior of *T. castaneum* in small landscapes (50 × 50 cm) varying in the abundance and size of flour patches and found that beetles moved more slowly and tortuously when the habitat was composed of many small as compared to few large patches. In a subsequent study, Romero et al. (2010) also found that patch condition influenced movement angles and velocities in the matrix near the patch boundary.

Finally, flour beetle movement is strongly influenced by the production of pheromones and semiochemicals (Campbell et al., 2022; Dissanayaka et al., 2020; Ó'Ceallacháin & Ryan, 1977; Suzuki & Sugawara, 1979). Males of *T. castaneum* and *T. confusum* produce the same aggregation pheromone 4,8‐dimethyldecanal (4,8 DMD) which is a short‐range (sub meter) attractant to both males and females. Females also produce pheromones attractive to males (Ó'Ceallacháin & Ryan, 1977; Olsson et al., 2006). Differences in pheromone production and responses by males and females may explain, in part, sex‐specific differences in dispersal propensity that were noted above. Both flour beetle species also produce chemical byproducts while feeding on flour that can act as repellants or attractants to conspecifics and heterospecifics (Alexander & Barton, 1943; Ghent, 1963; Loconti & Roth, 1953; Suzuki et al., 1975). Ghent (1963) found that *T. castaneum* was repelled while *T. confusum* was attracted to flour conditioned by conspecifics; the strength of those effects was lessened with increasing conspecific density. Later, Suzuki et al. (1975) found that volatile hydrocarbons produced by both species act as repellents to either species. Finally, Bullock et al. (2020) recently reported that flour conditioned by one species could affect life‐history traits of the other species (e.g., fecundity, development time). Studies attempting to link intra‐ and interspecific semiochemical production in flour beetles to movement behavior are scarce (but see Ghent, 1963), but these pheromones and semiochemicals likely play an important role in determining density‐movement relationships. Lastly, in dim or no‐light situations associated with stored grain products (and our experiments), visual cues are likely unimportant to flour beetle movement behavior.

Source material for our single‐species colonies of *T. castaneum* and *T. confusum* were collected from granaries in Louisiana, Indiana and Kentucky, USA during the fall–winter of 2017 and mixed to increase genetic diversity of the stock (for more details, see Harman, 2020). One hundred beetles of the same species were added to 500 mL containers with 200 g of organic whole wheat flour and nutritional yeast (19:1 mix) and placed in environmental chambers at 30 C, ~70% relative humidity and 12:12 day:night cycle. At weekly intervals, flour was sieved to isolate beetle eggs/larvae from adults and the juveniles were transferred to containers with fresh culture medium. When the next generation of adult beetles emerged, 100 individuals were transferred to a new container of flour/yeast. This procedure ensured that beetle densities per container would not reach a level where they were overexploiting their food resource. To further minimize differences among beetles used in our dispersal experiments, beetles were standardized for age and density. Adults from the colony that were ≤7 days post eclosion were transferred in groups of 50 individuals to 120 mL clear‐plastic containers with 10 g flour/yeast. One week later, we allowed for a 1‐week window of time in which those beetles could be used in experiments. As such, beetle adults were 14–21 days old in all trials and had ample opportunity to mate prior to the start of the experimental trials.

### 2.2 Within‐patch movement of flour beetles

#### 2.2.1 Experiment 1: Effects of conspecific density on within‐patch redistribution

**FIGURE 1:**
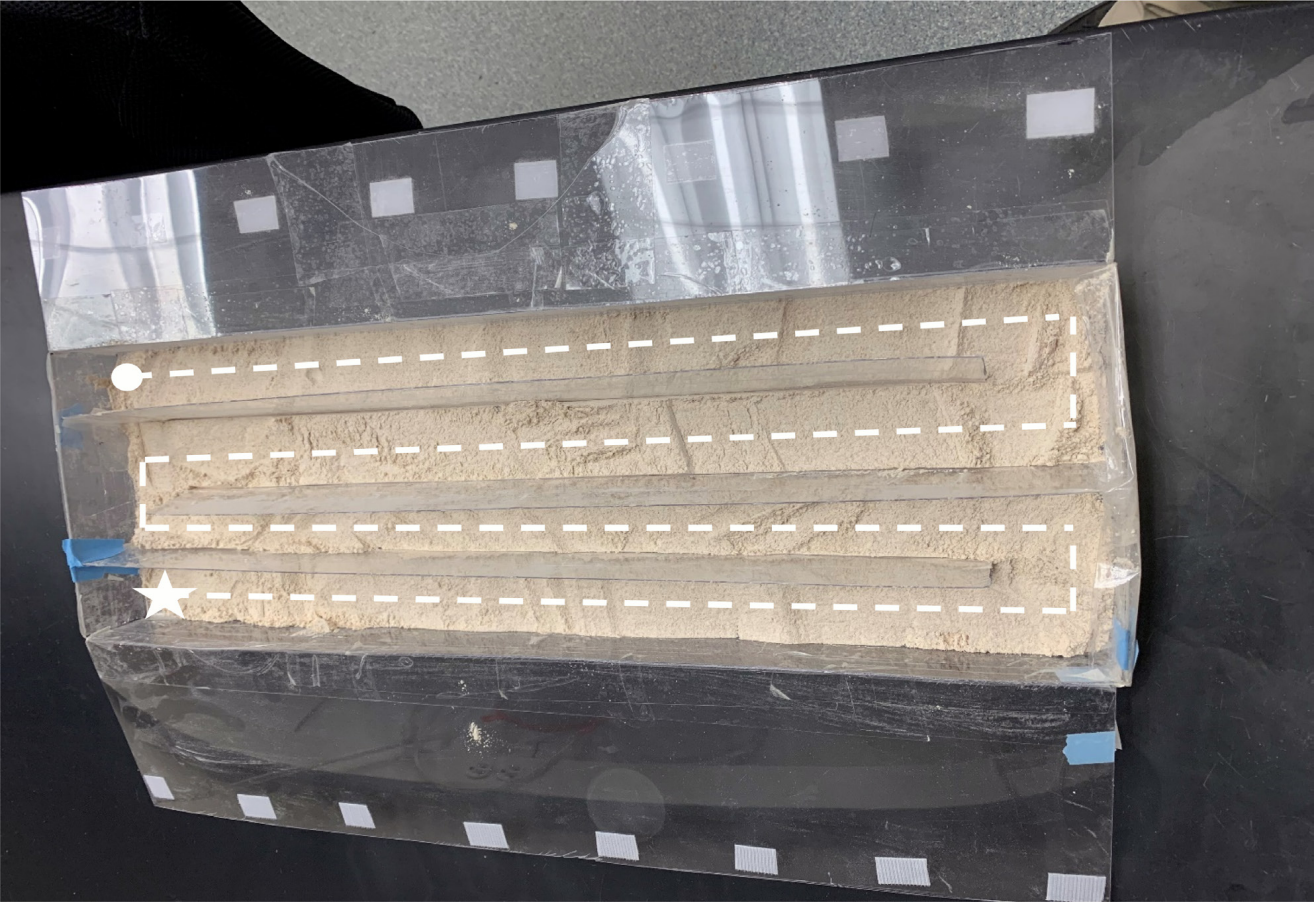
Experimental arena used to study the within‐patch movement of flour beetles. The patch consisted of four lanes (each 60 cm long × 5 cm wide) that were open at one end so that they were connected to the next adjacent lane. Beetles were released at one end of the arena (denoted by white star) and could traverse along the dashed pathway up to a distance of 240 cm (end of the fourth lane, white circle). Each lane had a 30 g layer of flour/yeast mix.

We manipulated the abundance of adult *T. confusum* and *T. castaneum* and assessed whether the within‐patch redistribution of each species can be well fit by a diffusion model and whether diffusion rates and mean displacements depend on the density of conspecifics. Our experimental patch consisted of a 60 cm × 20 cm box cut from 0.75 mm thick plastic sheeting held together with 25 mm wide clear shipping tape (Figure 1). Walls on the box were 5 cm tall. Within the box, we created four interconnected dispersal lanes using plastic sheeting that ran parallel to the long side of the box. Each lane had a 5‐cm opening at one end, allowing the beetles to pass through to the next lane (Figure 1). A thin layer of flour/yeast (30 g per lane) was spread across the floor of the rectangular box. This created a “linear” arena that allowed beetles to traverse via walking all lanes as if it were one 240 cm long patch. Through trial and error, we adopted this size of arena because it was extremely unlikely that a beetle would reach the end of the dispersal array in a one‐hour period (the duration of our trials). We note here that we never observed evidence of flight behavior in our experimental arenas.

For these single species trials, we released beetles at a density of 5, 10, 25, 50 and 100 adults. Based on a long‐term experiment with similar densities of both flour beetle species reared in enclosed containers with the same amount of flour as our dispersal arenas, we have found strong negative density dependence in the per‐capita population growth rate (Krivchenia et al., 2023). This result suggests that our range of densities is appropriate for assessing density effects on movement behavior.

Beetle adults were anesthetized with CO_2_ for 2 min and then released within a 5 cm^2^ area at the beginning of the first lane of the arena. The arena was sealed with a clear plastic lid and placed in an environmental chamber (same conditions as for the colonies; see above) for 1 h to allow adults to disperse. At the end of this time period, plastic partitions were placed in the linear patch to trap beetles within 15‐cm sections. Beetles in each 15‐cm section were removed and counted. Because males and females have been reported to exhibit different dispersal abilities (Ogden, 1970b; Ritte & Lavie, 1977), we also sexed all beetles from a subset of the trials.

Five to seven replicate trials for each density were conducted. Because of the time required to sex beetles, we only did this for 4–5 of the replicates per treatment combination. Also, owing to limitations on environmental chamber space, trials were staggered over a several month period of time (August–October, 2019). Trials for the various abundance × species combinations were distributed evenly among dates to avoid any temporal bias in results among treatments.

##### Statistical analyses

Using the method described by Okubo (1980), Kareiva (1983) and Turchin (1998), we fit a simple random‐diffusion model to the redistribution data for our within‐patch movement trials. The expectation of the model is that net movements should be normally distributed which can be evaluated with the model

$$N_{X}=Ae^{\left(-BX^{2}\right)}$$

where *N*_*X*_ is the density of beetles that have moved some distance, *X*. The diffusion model has the linear form ln(*N*_*X*_) = ln(*A*)–*BX* ^2^ and can be fitted using least‐squares regression. The model *R* ^2^ is a measure of the goodness‐of‐fit. Because of the short‐term nature of these trials (1 h), we deemed it unnecessary to account for mortality among beetles (Turchin & Thoeny, 1993). If redistribution data are well fit by the diffusion model, then the distribution of beetles at any time *t* provides an estimate of the diffusion coefficient, *D*. Here, *D* can be estimated as MSD/4 *t*, where MSD is the mean square displacement of released beetles at time *t* (Kareiva, 1983; Okubo, 1980; Turchin, 1998).

To maximize statistical power for the regression analyses, we used only the three highest density releases (25, 50 and 100 adults) for each species and summed counts per distance category from replicate trials. For the 15 cm distance categories within which beetles were lumped, we used the midpoint as the distance moved. Also, all distance categories beyond the last captured individual were omitted, with the exception that we included a zero for the next highest distance category beyond the one containing our furthest dispersed beetles. For ease of comparison between density treatments, we converted recaptures to a proportion. Separate least‐squares regression analyses for each density level were conducted in RStudio v 2023.06.1 (R Core Team, 2023) and package stats. Additional regression analyses were performed for males only and females only (for densities of 25 and 50 total individuals).

Given a good fit of the diffusion model to the redistribution of our beetles (see Section 3), we calculated the mean displacement per trial which can be used to estimate *D*, the diffusion rate. To simplify interpretation of our results, we chose to use mean displacement instead of *D* as the response variable in subsequent analyses of movement within a patch. However, we also report mean *D* per treatment as this is an important parameter for modeling beetle redistribution within a patch. First, we assessed whether males and females differed in their mean displacement (*ln*‐transformed to achieve normality and homogeneity of variances) using a general linear model (GLM). Separate analyses were performed for each species. Density level and sex were treated as categorical fixed factors and the model also included a density × sex interaction term.

Second, we used the full dataset, pooling males and females within a species (there was no significant difference between sexes; see Section 3), and used a similar GLM to test whether mean displacement (*ln*‐transformed) differed between flour beetle species and among density levels. Species and density were treated as fixed categorical factors in the model. For both tests, we used the *lm* function in *R* to fit the linear model, the car package to estimate Type III sums‐of‐squares and the emmeans package to estimate marginal means and to perform Tukey's pairwise *t* tests. Diagnostics for the GLM were assessed using Pearson residual plots (for homogeneity of variances) and quantile–quantile plots (for normality of residuals).

#### 2.2.2 Experiment 2: Heterospecific effects on within‐patch redistribution

Concurrent with the previous experiment, we also conducted releases of adult *T. castaneum* and *T. confusum* together in the same arena (see Figure 1) and quantified the mean displacement of each species. Total beetles released were either 10, 50 or 100 (1:1 ratio of each species). There were 8, 5 and 6 replicate trials for each of these density levels, respectively. Beetles were anesthetized, gently mixed together and then released at one end of the arena. The procedure for executing these trials was exactly the same as for the previous experiment.

##### Statistical analyses

To assess heterospecific effects on species mean displacement, we compared *ln* mean displacement for the following three situations: (1) species *i* when alone at density *x*, (2) species *i* in the presence of species *j* with both at the same density *x* and (3) species *i* when alone at density 2*x*. For example, we compared the displacement of *T. castaneum* in trials with 5 *T. castaneum* (situation 1), 5 *T. castaneum* + 5 *T. confusum* (situation 2) and 10 *T. castaneum* alone (situation 3). If displacement differed between situations 1 and 2, that would indicate the heterospecific (in this case, *T. confusum*) affected *T. castaneum* displacement. A comparison of case 1 and 3 would assess whether increased conspecific density affects *T. castaneum* movement, and a comparison of case 2 and 3 would indicate whether the identity of the heterospecific matters (i.e., the effect of the other species is not simply due to increase in total beetle abundance). A separate GLM was performed for each mixed‐species density release, with situation treated as the predictor variable.

## 3 RESULTS

### 3.1 Experiment 1: Effects of conspecific density on within‐patch redistribution

**FIGURE 2:**
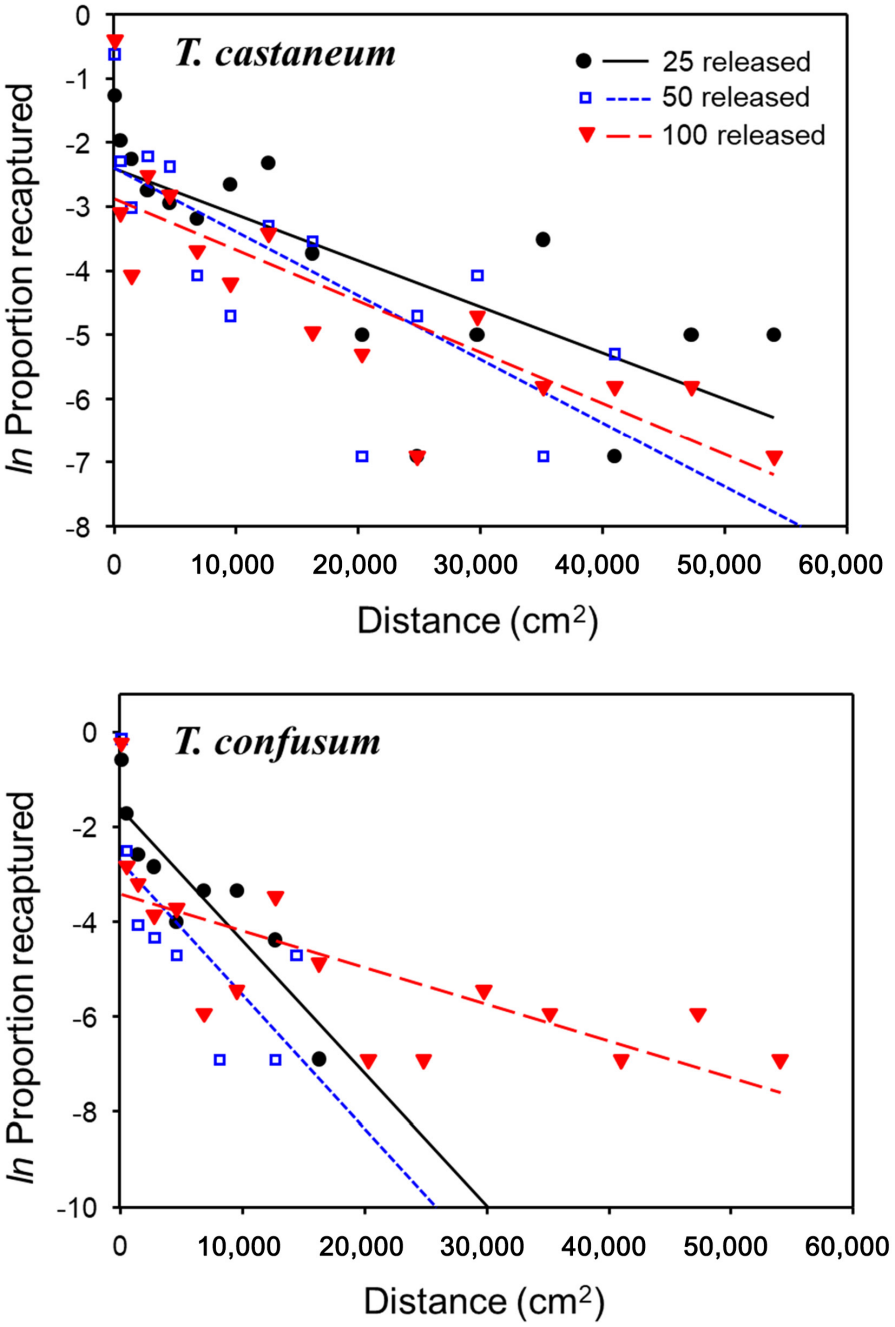
The *ln* proportion of *T. castaneum* adults (a) and *T. confusum* adults (b) recaptured 1 h after release into the arena in Figure 1. Only one species was released in each trial at a density of either 25, 50 or 100 adult beetles. For each density level, symbols represent the combined recaptures from all 5–7 replicates per density level (as a proportion of the total released). Lines for each density level are fit by least‐squares regression (see Table 1 for statistical results).

Redistribution within a patch was well described by a simple diffusion model for both *T. castaneum* and *T. confusum* (Figure 2). The fit of the diffusion model to the different release densities was similar on average between species: *R* ^2^ ± SE was 0.65 ± 0.01 for *T. castaneum* and 0.61 ± 0.10 for *T. confusum* (Table 1). When analyzed separately, the redistribution of males and females of each species was also well described by the diffusion model, judging by model *R* ^2^ (Table 1). In all cases, adding a quadratic term to the regression model did not improve model fit (i.e., no significant distance^2^ effect). Because of the consistently good fit of the simple diffusion model to the redistribution of beetles, hereafter we used the mean displacement (in cm) and the diffusion rate, *D* = MSD/4 *t* (Kareiva, 1983; Okubo, 1980; Turchin, 1998) as our metrics for each species, density level and replicate.

**TABLE 1:** Least‐squares regression results for the relationship between squared displacement distance (cm^2^) and the *ln* proportion recaptured at a particular distance (in 15‐cm‐long blocks).

Species	Sex^a^	Density	*R* ^2^	df	*F*	*p*
*T. castaneum*	Both	25	.619	1,14	22.72	.003
	Both	50	.652	1,13	24.34	.003
	Both	100	.664	1,14	27.60	<.001
	Males	25	.539	1,11	12.84	.004
	Males	50	.284	1,5	1.98	.218
	Females	25	.361	1,12	6.78	.023
	Females	50	.488	1,7	6.67	.036
*T. confusum*	Both	25	.793	1,7	26.85	.001
	Both	50	.487	1,6	5.69	.054
	Both	100	.529	1,14	15.74	.001
	Males	25	.495	1,4	3.92	.119
	Males	50	.306	1,14	6.16	.026
	Females	25	.723	1,4	10.42	.032
	Females	50	.468	1,8	7.03	.029

*Note*: Separate analyses were conducted for *T. castaneum* and *T. confusum*, release densities and sex (males, females or both combined).

^a^ Release density is based on the combined number of males and females. Because the sex ratio is close to 1:1, the actual numbers of males or females released is approximately ½ the total released.

**FIGURE 3:**
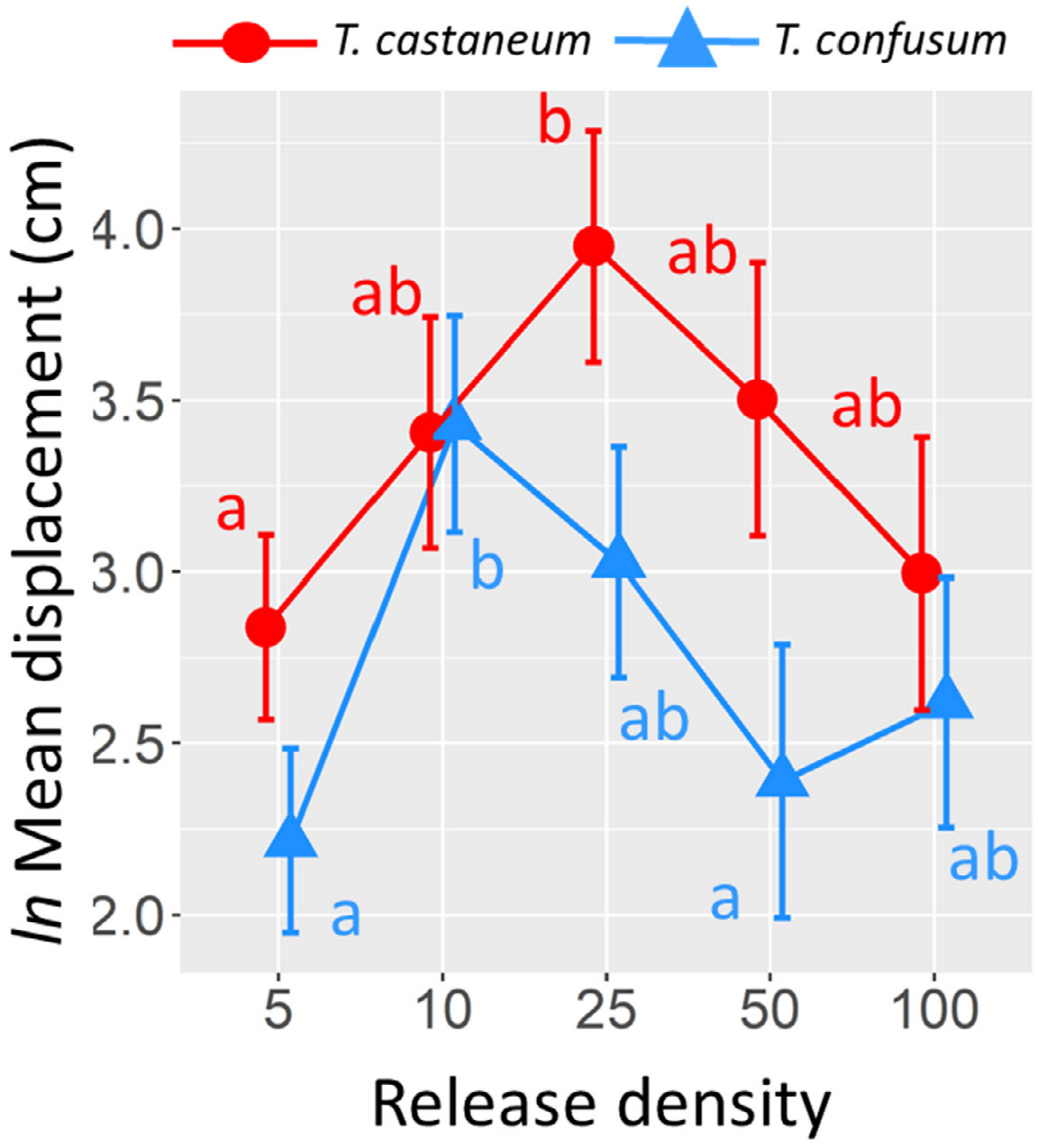
For single‐species trials, the *ln* mean displacement (at 1 h) ± 1 SE of *T. castaneum* and *T. confusum* within a patch in response to different conspecific densities. Statistical results for the GLM are reported in Table 3. For each species, different letters denote treatment means that are significantly different based on Tukey's pairwise *t*‐tests (*p* ≤ .05).

Among trials with *T. castaneum* alone, there was no difference in the mean displacement (*ln*‐transformed) between females and males, irrespective of release density (Table 2). On average, males and females differed by only 1% in their displacement (back‐transformed marginal mean and 95% CIs; females: 26.6 cm [10.8, 65.4], males 26.3 cm [10.5, 66.0]). Similarly, there was only an 11% difference in the displacement between female and male *T. confusum* (females: 13.7 cm [8.9, 21.1], males 15.2 cm [10.6, 21.5]). This difference was not statistically significant (Table 2). Subsequent analyses of within‐patch movement used the combined number of males and females for each species. In single‐species trials, *T. castaneum* displaced 82% farther than *T. confusum* (marginal means and CIs; *T. castaneum*: 28.2 cm [20.5, 38.5], *T. confusum*: 15.5 [11.4, 20.9]) and this difference was significant (Table 3; Figure 3). The diffusion rate, *D*, was estimated to be 198.8 cm^2^/h for *T. castaneum* and 60.1 cm^2^/h for *T. confusum*.

**TABLE 2:** ANOVA results from a general linear model (GLM) in which mean displacement (*ln*‐transformed) was the response variable and conspecific density (5, 10, 50 and 100), sex (male or female) and density × sex were predictor variables.

Species	Factor	Sums‐of‐squares	df	*F*	*p*
*T. castaneum*	Intercept	352.75	1	678.608	<.001
	Density	6.71	3	4.303	.011
	Sex	1.31	1	2.518	.121
	Density × Sex	0.58	3	0.374	.772
	Residuals	18.71	36		
*T. confusum*	Intercept	262.72	1	383.453	<.001
	Density	1.21	3	0.591	.625
	Sex	0.09	1	0.125	.726
	Density × Sex	0.72	3	0.351	.789
	Residuals	23.30	34		

*Note*: Separate analyses were conducted for *T. castaneum* and *T. confusum*. Type III sums‐of‐squares are reported.

**TABLE 3:** For single species trials, GLM results for the effect of species (*T. castaneum*, *T. confusum*), conspecific density (5, 10, 25, 50 and 100) and species × density on *ln* mean displacement (cm) of flour beetles after 1 h.

Factor	Sums‐of‐squares	df	*F*	*p*
Intercept	614	1	773.576	<.001
Species	6	1	7.586	.008
Density	11	4	3.562	.011
Species × Density	3	4	0.821	.517
Residuals	49	62		

*Note*: Type III sums‐of‐squares are reported.

Mean displacements for both *T. castaneum* and *T. confusum* were highest at intermediate densities (Figure 3). There was no species × density interaction (Table 3), indicating that there was no significant difference in response by the two species to density. A comparison between density levels (after controlling for species) indicated that displacement at a conspecific density of 5 individuals was 3.4 times lower than the displacement at a density of 10 (back‐transformed data; *t* = 2.98, *p* = .032) and 2.3 times lower than at a density of 25 (*t* = 3.16, *p* = .020). All other pairwise differences were not statistically significant. We note that treating density as a continuous variable and including a quadratic term (density^2^) in the model revealed a significant quadratic relationship with density for *T. castaneum* (*ln* mean displacement = 24.22 + 0.96[density] – 0.01[density^2^], *R* ^2^ = 0.14, *p* ≤ .05 for both density terms) but neither a linear (*ln* mean displacement = 27.21–0.013[density], *R* ^2^ = 0.05, *p* > .05) nor quadratic relationship for *T. confusum* (*ln* mean displacement = 30.47–0.42[density] + 0.002[density^2^], *R* ^2^ = 0.07, *p* > .05).

### 3.2 Experiment 2: Heterospecific effects on within‐patch redistribution

**FIGURE 4:**
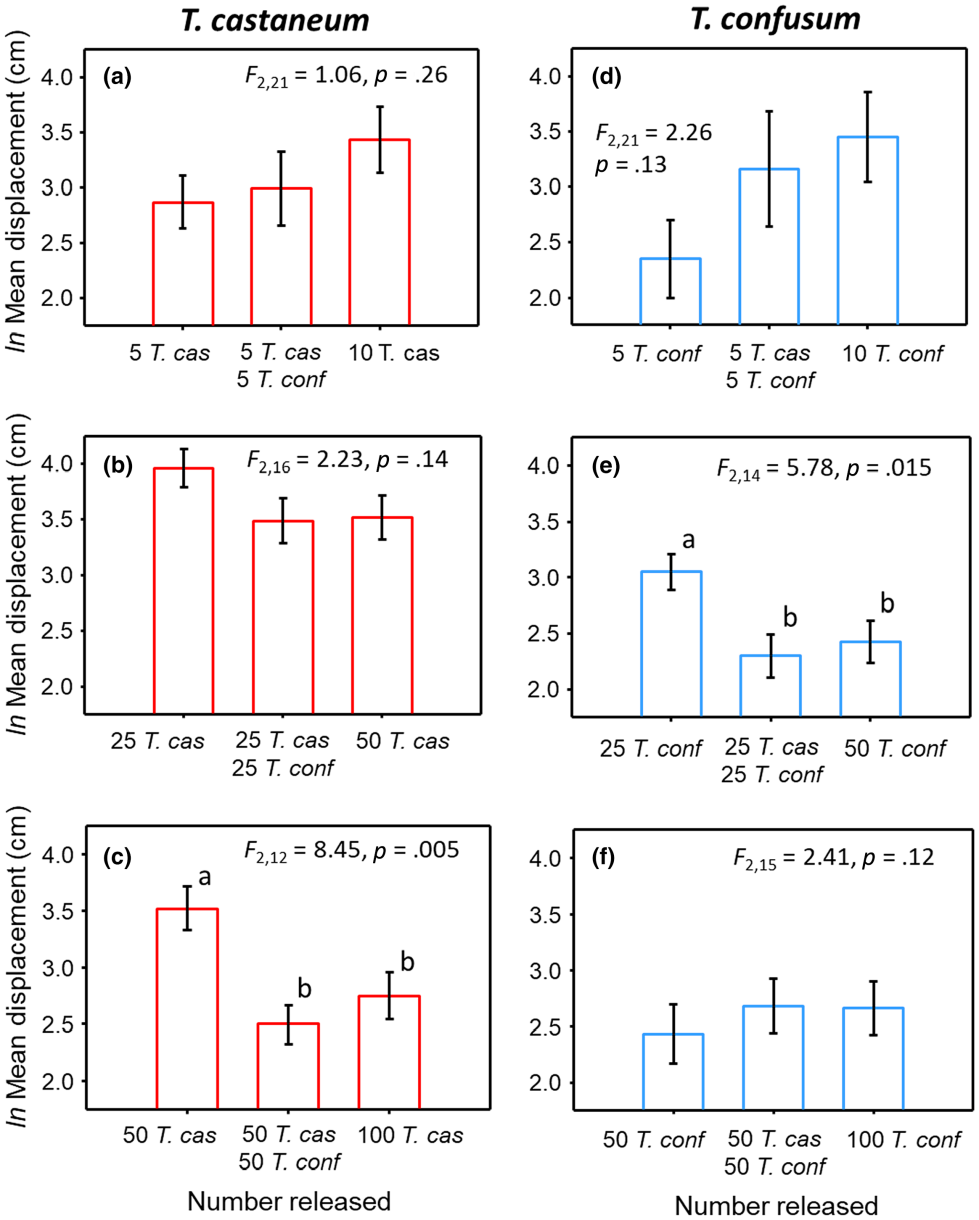
*ln* mean displacement ±SE for *T. castaneum* (*T. cas*; left column) and *T. confusum* (*T. con* right column) when occurring alone at an abundance of *x*, together with the other species each at an abundance of *x*, and alone at an abundance of 2*x*; where *x* = 5 (a, d), 25 (b, e) or 50 (c, f) adult beetles. *F*‐statistics and *p* values from separate GLMs for the effect of number released are reported. In cases where *p* ≤ .05, Tukey's pairwise *t*‐tests were performed and lower‐case letters denote treatment means that are significantly different (*p* ≤ .05).

For both *T. castaneum* and *T. confusum*, there was evidence in only one of three density comparisons that the presence of one beetle species affected the mean displacement of the other beetle species (Figure 4). When 50 *T. castaneum* were released alone, their mean displacement was 2.8 times greater (based on back‐transformed means) than when the same number of *T. castaneum* were present with an equal number of *T. confusum* (Figure 4c). The mean displacement in this mixed species group (with a total of 100 beetles) was not different from the mean displacement of 100 *T. castaneum* alone, suggesting that the *T. confusum* effect was primarily a density effect. A similar result was found for *T. confusum* when released at an abundance of 25 individuals (Figure 4e). Mean displacement of the 25 *T. confusum* was 2.1 times higher when *T. confusum* was alone than when it occurred with an equal number of *T. castaneum*. Although not statistically significant, at our lowest density releases of *T. castaneum* or *T. confusum*, there was a tendency for the presence of the heterospecific and the two‐times density situation to promote greater mean displacement (Figure 4a,d). This result likely reflects the curvilinear relationship between density (when alone) and mean displacement as shown in Figure 3.

## 4 DISCUSSION

As noted two decades ago by Morales and Ellner (2002), the main challenge for scaling up movement patterns to population processes resides in the complexities of individual behavior. Movement can be highly species specific (Markow & Castrezana, 2000; Ziegler, 1977), depend on the sex, production of pheromones and semiochemicals, life stage, mating status or life‐history traits of the species (Beirinckx et al., 2006; Bellamy & Byrne, 2001; Campbell et al., 2022; Cronin et al., 2000; Markow & Castrezana, 2000; Sappington & Showers, 1992), vary in non‐linear ways with respect to conspecific density (Amarasekare, 1998; Harman et al., 2020), and depend on the density of interacting species (e.g., competitors and predators) (Dallas et al., 2019, 2020; Hakkarainen et al., 2001; Sih et al., 1992); in addition to being affected by environmental conditions (e.g., temperature, wind speed, light levels) and characteristics of the patch (e.g., size, isolation) and landscape (e.g., matrix composition) (Turchin & Omland, 1999). Although the redistribution of both *T. castaneum* and *T. confusum* within a patch were well described by a simple random‐diffusion model, these two congeneric species with very similar physical appearances differed substantially in their mean displacements. The former species displaced 82% farther and had a diffusion rate that was 3.3 times faster than the latter species. However, movement rates for both species exhibited a humped‐shaped relationship with respect to conspecific density, although peak movement rates differed between species. Finally, for both species, the addition of a heterospecific competitor had the same effect as the addition of conspecifics, suggesting that the propensity to move is a generic response to potential competitors. We discuss these results in more detail below, along with how within‐patch movement may scale up to affect the spatial dynamics of the system.

Beginning with Dobzhansky and Wright (1943) and continuing through the analysis of herbivore movement by Kareiva (1983), the redistribution of a wide range of taxa, both invertebrates and vertebrates, have been remarkably well fit by a diffusion model framework (e.g., Abramson et al., 2006; Antonelli et al., 1993; Bodino et al., 2020; Cronin et al., 2001; Fortin et al., 2013; Skalski & Gilliam, 2000; Turchin & Thoeny, 1993). When released in a homogeneous environment at three different densities, both *T. castaneum* and *T. confusum* exhibited an exponential decay in their density with respect to distance from the release point. A simple diffusion model explained over 60% of the variation in recaptures‐with‐distance and we found no evidence for a curvilinear relationship that would be expected if leptokurtosis (fat‐tails in the distribution) was present (Cronin et al., 2000; Fraser et al., 2001). This result provides confirmation that our one‐dimensional arenas, used for species that live in a two‐ or three‐dimensional world, yields a pattern of recaptures‐with‐distance that is consistent with the findings for many other species. However, we do note that our estimated mean‐displacement distances, 28.2 cm/h for *T. castaneum* and 15.5 cm/h for *T. confusum*, may be greater than would be found if unbounded two‐dimensional arenas were used. In comparison to our study, Morales and Ellner (2002) found strong evidence for fat‐tailed dispersal kernels for *T. confusum* released in a heterogeneous micro landscape, and that was attributed to heterogeneity in individual dispersal behavior.

Previous laboratory studies of *Tribolium* movement focused on either heterogenous landscapes (a flat surface with discrete patches of flour) (Naylor, 1959, 1961) or a series of interconnected patches (Dallas et al., 2019, 2020; Melbourne & Hastings, 2009; Ogden, 1970a, 1970b; Prus, 1963; Ritte & Lavie, 1977; Ziegler, 1978) and generally focused on beetles walking between patches of flour. Thus, direct comparisons are not possible. However, high rates of within‐patch diffusion are likely to lead to higher rates of emigration (Cronin et al., 2019; Ovaskainen, 2004). Therefore, the expectation is that *T. castaneum* would have a higher emigration rate (via walking) than *T. confusum*, which was supported by several (Dallas et al., 2020; Krivchenia et al., 2023; Ziegler, 1978) but not all (Ogden, 1970b) mesocosm studies. In the field, where monitoring of the dispersal of stored‐grain pests is conducted using pheromone‐baited traps, flight‐capable *T. castaneum* can disperse on the order of kilometers, much farther than the non‐flying *T. confusum* (Daglish et al., 2017; Holloway et al., 2020; Park, 1934; Ridley et al., 2011). In the future, an experimental study similar to the design of Turchin and Thoeny (1993) for the southern pine beetle could be used to assess whether redistribution of *Tribolium* at large scales can be described by diffusion models.

Results have been mixed as to whether male and female flour beetles of the same species had different movement or emigration rates. Although ours and several other studies found no evidence for differences in movements of males and females (Krivchenia et al., 2023; Ziegler, 1977, 1978), some studies found that males emigrated more readily than females (Hagstrum et al., 1998; Ogden, 1970b) while others found the opposite result (Matsumura & Miyatake, 2015). In the broader ecological literature, there appears to be no one sex that is the better or more likely disperser (Beirinckx et al., 2006; Bellamy & Byrne, 2001; Bowler & Benton, 2009; e.g., Cronin et al., 2000). For both *Tribolium* species, which produce aggregation pheromones, it remains unexplored whether the sex ratio influences male and female movement behavior.

An intuitive expectation is that movement or emigration rates should increase as density increases – a means to lessen the impacts of intraspecific competition (Amarasekare, 2004; Harman et al., 2020). In their literature review, Harman et al. (2020) found that the form of the relationship between density and the rate of emigration was quite variable. Although density‐independent and positive density‐dependent forms were most prevalent, accounting for two‐thirds of all cases, negative density dependence (25% of cases) and nonlinear forms (including humped‐ and u‐shaped relationships; 9% of cases) have been reported. Studies with *Tribolium* have reported positive density‐dependent emigration (Naylor, 1965; Ogden, 1970b; Ziegler, 1978). Aggregation pheromones, such as those produced by *Tribolium*, are thought to have evolved in species that experience an Allee effect; that is, positive density dependence in per‐capita population growth (Halliday & Blouin‐Demers, 2016; Wertheim et al., 2004). Although unexplored, aggregation pheromones could also affect the density–movement relationship. One possible expectation is that at lower densities, as density and the release of aggregation pheromones increases, movement becomes increasingly suppressed. In contrast, at higher densities, the increased intensity of competition and production of repellent semiochemicals (Ghent, 1963; Loconti & Roth, 1953; Suzuki et al., 1975) become paramount, resulting in higher movement rates. Across the entire density range, this would result in a u‐shaped density–movement relationship. In contrast, we found that for both flour beetle species, peak displacement or diffusion rate occurred at some intermediate density. Just as with emigration, a humped‐shaped relationship could arise when the benefits of living in small and large groups are greater than in intermediate‐sized groups. At low density, the population may be less noticeable to predators and resources are likely to be most abundant; whereas at high density, defense against predators may be more effective or resources may be more easily or efficiently exploited (Harman et al., 2020; Jacob et al., 2016). Given that *Tribolium* are cannibalistic against juvenile life stages (Benoit et al., 1998; Park et al., 1974; Rich, 1956), it seems unlikely that beetles would benefit in the long‐term from remaining in high‐density situations. However, in our experimental setup, only adults were present so that the immediate threat of cannibalism did not exist. At this point, we cannot explain this unusual behavior.

A dispersal‐competition tradeoff is a well‐known mechanisms of coexistence for species living in a patchy environment (Chesson, 2000; Tilman, 1994). But what if one competitor directly influences the movement of the other competitor? If the superior competitor promotes higher rates of dispersal or strengthens the form of density‐dependent dispersal in the inferior competitor, it could strengthen the competition‐dispersal tradeoff and potentially increase the likelihood of coexistence (Chesson, 2000). On the other hand, if the competitor causes negative density‐dependent dispersal in the other species, or if the presence of the inferior competitor promotes higher dispersal in the superior competitor, it could weaken or eliminate the tradeoff.

In this study, we found that one species of flour beetle did impact the within‐patch movement rates of the other species, but it generally changed with density. For *T. castaneum* in the highest density scenario of 50 individuals released, adding another 50 *T. castaneum* or 50 *T. confusum* had the same effect – displacement was reduced by approximately one half. The same thing occurred for *T. confusum* released at a lower density. It suggests that, in both cases, the identity of the beetle species did not matter to the movement of the *Tribolium* species – displacement distance (or diffusion rate) was negatively related to density regardless of the species. At the lowest densities, we also observed that species identity did not matter, but that movement was positively related to total density (although the effect was only significant for *T. confusum*). The reason for the switch in response to density was likely related to the humped‐shaped density–movement curves reported in Figure 3. At lower densities, movement rates increased with density, and at higher densities, movement rates declined with density. Given that both *Tribolium* species produce similar aggregation pheromones and repellent semiochemicals (Alexander & Barton, 1943; Loconti & Roth, 1953; Suzuki et al., 1975), it may not be surprising that beetle movement behavior is similar in response to the presence of conspecifics or the other beetle species.

Other studies that have explicitly tested whether a species affects the dispersal behavior of its competitors are quite rare. Using the same flour beetle system as us, Dallas et al. (2020) found that *T. castaneum* dispersal was reduced in the presence of *T. confusum*. Similarly, De Meester et al. (2015) was able to demonstrate with a community of nematodes that the presence of competitors caused one species of nematode to accelerate its time to dispersal, a measure of dispersal ability according to the authors. Indirect evidence comes from cases of competitor‐induced changes in morphology – for example, for two species of salt‐marsh planthoppers, interspecific crowding caused the increase in production of macropters, long‐winged individuals capable of long‐distance flight (Denno & Roderick, 1992). Similar result was found in Matsumura and Suzuki (2003). However, with so few studies available, it is difficult to determine whether competitor effects on dispersal behavior are common. One potentially fruitful avenue for investigating this problem is with range‐expanding species. Studies of range expansion by native or invasive species provide evidence that resident competing species reduce the rate of range expansion (Svenning et al., 2014). Escape from competitors in their native range may alternatively facilitate range expansion by invasive species. A fertile area of research would be to dissect whether changing expansion rates are due to changes in population growth, dispersal behavior or some combination of both. Finally, just as there is growing evidence that predators affect the dispersal behavior of their prey, a trait‐mediated effect (e.g., Hakkarainen et al., 2001; Sih et al., 1992), we expect that additional studies will reveal the diversity and prevalence of competitor‐induced dispersal behavior.

Finally, this study focused only on the effects of adult beetle density on the movement of adult conspecific and heterospecific beetles. The effects of the larval environment – either the larval density or species composition in which the adults completed their juvenile development, or larval density and species composition contemporaneous with the adults – on adult beetle movement were left unexplored. However, it is well known that an adult's propensity to disperse can be influenced by the conditions of its larval environment. For example, for many insect species, higher larval densities lead to a greater production of macropterous (long‐winged) adults (e.g., Denno et al., 1985; Fujisaki, 1986; Herzig, 1995; Poniatowski & Fartmann, 2011). As Endriss et al. (2019) demonstrated with *T. castaneum*, a poor larval environment can affect not only the phenotype of the adult but also that of its neighbors; both of which can influence an individual's movement behavior. If resource competition also takes place between juveniles and adults, as is the situation with *Tribolium*, then juvenile density is also likely to affect adult movement and dispersal. Finally, we are aware of no studies that have examined species composition in the juvenile stage and its effects on adult movement. We suggest that future research on *Tribolium* movement behavior should focus on prior and current habitat conditions, including the effects of juvenile density, larval interspecific competitors and even the presence of natural enemies.

### 4.1 Implications for population dynamics

Understanding the context‐dependency of species dispersal behavior is crucial to predicting spatial spread of populations, minimum patch size, metapopulation dynamics, species persistence and coexistence (Hanski, 1999; Hengeveld, 1989; Turchin, 1998), and has indisputable benefits to conservation and management efforts. Classic reaction–diffusion models predict a linear relationship between time and the radius or square‐root of the area occupied by a range‐expanding species (Hastings, 1996; Okubo, 1980; Shigesada et al., 1995). Nonlinearities in the relationship can be caused by Allee effects during the early stages of population establishment and density dependence in population growth. Models that include the presence of an interspecific competitor or predator using a Lotka‐Volterra or similar framework can also cause nonlinearities in the rate of population spread (Hastings, 1996; Okubo et al., 1989). However, these models do not consider the possibility that the diffusion rate can vary with conspecific density or depend on the presence of interacting species. Several studies have experimentally examined the spatial spread of *Tribolium* in patchy one‐dimensional mesocosms (Dallas et al., 2020; Melbourne & Hastings, 2009; Morales & Ellner, 2002). In Morales and Ellner (2002), model fit to the rate of population spread of *T. confusum* was drastically improved by accounting for temporal variability in movement behavior, whereas for Melbourne and Hastings (2009), fat‐tailed dispersal kernels, reflecting individual heterogeneity in *T. castaneum*, improved model fit. In their mixed‐species trials, Dallas et al. (2020) found that individual‐level variability in dispersal behavior and presence of an interspecific competitor can explain variability in spatial spread. Based on our study, we expect that variability in the spatial spread of *Tribolium* would be further increased when movement behavior is also a nonlinear function of conspecific and heterospecific density. Extending our work to range‐expanding species, we believe it would be beneficial for future studies to focus on how variation in conspecific and interacting species densities along a transect from the range interior to the edge are influencing movement behavior.

Our study focused on species movement within small homogeneous habitat patches. Practical limitations often necessitate measuring and analyzing movements at small spatial scales (Kareiva & Andersen, 1988; Morales & Ellner, 2002). Scaling up these findings to heterogeneous landscapes would be invaluable to pest managers and conservation biologists. Random walk and diffusion approximations have been successful mechanistic models used to describe animal movement at small scales but can give unrealistic predictions when extrapolating to larger scales (Fryxell et al., 2008; Morales & Ellner, 2002; Turchin, 1996). Responses to habitat boundaries (Ovaskainen, 2004; Stamps et al., 1987), encounters with different matrix compositions (Cronin & Haynes, 2004; Roland et al., 2000), engaging in different activities (e.g., mate finding, nest building, predator avoidance; Fryxell et al., 2008; Ryan & Cantrell, 2015) and the physiological state of the organism (Bell, 1991) can change movement behavior. Accurate predictions at the landscape scale will require models and experiments that can accommodate the complexities of individual movement behavior (Cronin & Reeve, 2005; Lima & Zollner, 1996). One significant benefit of scaling up movement is that behavior‐based approaches (using models or empirical research) can be used to quantify functional connectivity within real landscapes; an important key to understanding metapopulation and metacommunity dynamics (Baguette et al., 2012; Ovaskainen et al., 2008; Richter‐Boix et al., 2007). Our long‐range goal with the *Tribolium* system is a theoretical and empirical research program that scales up from patches to landscapes, accounting for semi‐permeable boundaries, hostile matrix habitats, multiple habitat patches and interactions with competitors and predators.

## AUTHOR CONTRIBUTIONS

**James T. Cronin:** Conceptualization (equal); formal analysis (lead); funding acquisition (equal); investigation (lead); methodology (lead); writing – original draft (lead); writing – review and editing (lead). **Jerome Goddard II:** Conceptualization (equal); formal analysis (equal); funding acquisition (equal); investigation (supporting); writing – review and editing (supporting). **Aaron Krivchenia:** Data curation (supporting); investigation (supporting); writing – original draft (supporting); writing – review and editing (supporting). **Ratnasingham Shivaji:** Conceptualization (equal); formal analysis (supporting); funding acquisition (equal); methodology (equal); writing – original draft (supporting); writing – review and editing (supporting).

## CONFLICT OF INTEREST STATEMENT

The authors declare no competing interests.

## Data Availability

Data and R code used in the analyses of these data are available from the Dryad Digital Repository (https://datadryad.org/stash/share/7Vlx3r_ac3_eaocHn7fv7v4V2klWVbXZwa6zihQCAgE).
